# Supplementary material for: Impact of Proactive Therapeutic Drug Monitoring on Infliximab Maintenance Therapy and Clinical Outcomes in Pediatric Inflammatory Bowel Disease: A Randomized Controlled Trial and Review of Literature
Source: Gastro Hep Adv. 2026 Apr 7;5(6):100953. doi: 10.1016/j.gastha.2026.100953 (PMC13158754; doi:10.1016/j.gastha.2026.100953)
Supplement: Supplementary Table 1 [file mmc1.pdf]

Supplemental Table 1. Post Optimization Characteristics

Characteristics	Post-Optimization phase	Post-Maintenance SOC Group (n = 19)	Post-Maintenance pTDM Group (n=21)
Treatment optimization, n (%)	29 (58)	4 (21)	5 (24)
Type of optimization, n (%)			
IMM	1 (3)	0	1 (20)
Infliximab dose only	13 (44)	3 (75)	4 (80)
Infliximab frequency only	5 (17)	0	0
Infliximab dose + frequency	8 (28)	0	0
IMM + Infliximab dose	2 (7)	1 (25)	0
Mean number of optimizations per patient, n	1.1 ± 0.3	1.25 ± 0.5	1 ± 0
Reason for optimization, n (%)			
Symptoms only	1 (4)	1 (25)	1 (20)
Abnormal labs only	0	0	0
Symptoms + abnormal labs	0	1 (25)	0
Abnormal infliximab assay	17 (63)	0	3 (60)
Abnormal assay + symptoms	3 (11)	2 (50)	1 (20)
Abnormal assay + symptoms + labs	5 (18)	0	0
Weight gain	1 (4)	0	0
