# Supplementary material for: Impact of Proactive Therapeutic Drug Monitoring on Infliximab Maintenance Therapy and Clinical Outcomes in Pediatric Inflammatory Bowel Disease: A Randomized Controlled Trial and Review of Literature
Source: Gastro Hep Adv. 2026 Apr 7;5(6):100953. doi: 10.1016/j.gastha.2026.100953 (PMC13158754; doi:10.1016/j.gastha.2026.100953)
Supplement: Supplementary Table 2 [file mmc2.pdf]

Supplemental Table 2. Characteristics Pre and Post Optimization phase

Characteristics	Pre-Op	Post-Op	Mean Diff/SE	95% CI	*p*
Weight (kg)	63.50±24.00	66.16±24.49	2.56±5.55	-0.79-4.44	**.006***
BMI (kg/m2)	23.11±8.26	24.90±7.47	1.79±4.53	0.23- 3.34	**.026***
Hemoglobin (g/dL)	13.04±1.45	12.92±1.49	0.12±0.84	-0.15-0.38	.391
Albumin (g/dL)	4.24±0.41	4.28±0.31	0.04±0.38	-0.16-0.08	.513
C-reactive protein (mg/dL)	5.51±6.02	5.33±3.34	0.18±6.62	-1.96-2.33	.863
ESR	12.38±11.78	11.08±9.07	1.30±9.58	-1.76-4.36	.396
PCDAI/PUCAI	9.13±9.53	6.31±6.88	2.82±5.46	1.06-4.56	**.002***
Clinical remission n (%)	21/40 52.5%	28/40 70.0%			.168
Dose infliximab (mg/kg)	7.81±2.38	9.24±2.06	1.43±2.55	0.61-2.24	**.001***
Frequency of infliximab (weeks)	7.63±0.77	6.90±1.00	0.75+0.96	0.42-2.24	**≤.001***
Abnormal infliximab assay, n (%)	24/40 60.0%	14/40 35.0%			**.043***
Infliximab trough conc (ug/dL)	6.04±3.82	9.31±3.58	+3.27±4.00	1.99-4.55	**≤.001***
Abnormal infliximab trough, n (%)	19/40 47.5%	1/40 2.5%			**≤.001***
Infliximab antibody (ng/mL)	58.03±90.99	49.88±70.04	8.15±73.08	-15.22-31.52	.485
Abnormal infliximab antibody, n(%)	18/40 45.0%	13/40 32.5%			.359
Patient QOL survey (scale 5-25, 5 = best health)	6.31±1.76	5.92±1.09	0.39±1.41	-0.07-0.84	.096
Patient well being scaling score (100 = best health)	88.10±17.15	89.95±11.01	1.85-14.16	2.68-6.38	.414
Parent reported QOL survey (scale 5-25, 5 = best health)	6.69±2.21	6.00±1.44	0.069±1.47	0.13-1.25	**.017***
Parent reported well being scaling score (100 = best health)	86.50±18.01	92.21±8.69	5.71±12.52	0.86-10.57	**.023***
