# Supplementary material for: Impact of Proactive Therapeutic Drug Monitoring on Infliximab Maintenance Therapy and Clinical Outcomes in Pediatric Inflammatory Bowel Disease: A Randomized Controlled Trial and Review of Literature
Source: Gastro Hep Adv. 2026 Apr 7;5(6):100953. doi: 10.1016/j.gastha.2026.100953 (PMC13158754; doi:10.1016/j.gastha.2026.100953)
Supplement: Supplementary Table 3 [file mmc3.pdf]

Supplemental Table 3. Adverse Events

Event, n (%)	Optimization phase (N = 51)	Maintenance phase SOC (N = 18)	Maintenance phase TDM (N = 21)	Related to Infliximab
Perirectal abscess			1 (4.8)	N
Pharyngitis			1 (4.8)	U
Pilonidal cyst			2 (9.5)	N
Apraxia			1 (4.8)	N
Folliculitis	1 (2)			U
Depression	1 (2)	1 (5.5)		N
COVID	1 (2)			U
Neutropenia		1 (5.5)		N
Headache	1 (2)			U
Tremors		1 (5.5)		U
Psoriasis	1 (2)			Y

Y = yes, N = no, U = unknown
