# Supplementary material for: Impact of Proactive Therapeutic Drug Monitoring on Infliximab Maintenance Therapy and Clinical Outcomes in Pediatric Inflammatory Bowel Disease: A Randomized Controlled Trial and Review of Literature
Source: Gastro Hep Adv. 2026 Apr 7;5(6):100953. doi: 10.1016/j.gastha.2026.100953 (PMC13158754; doi:10.1016/j.gastha.2026.100953)
Supplement: Extended PDF [file mmc4.pdf]

Gastro Hep Advances 2026;5:100953

ORIGINAL RESEARCH—CLINICAL

Impact of Proactive Therapeutic Drug Monitoring on Infliximab Maintenance Therapy and Clinical Outcomes in Pediatric Inflammatory Bowel Disease: A Randomized Controlled Trial and Review of Literature

Kristen Cares,[1] Ron Thomas,[2] Amy Stolinski,[3] Jacqueline Parker,[3] and Mohammad El-Baba[1]

[1]*Division of Pediatric Gastroenterology, Children's Hospital of Michigan, Detroit, Michigan;* [2]*Department of Pediatrics, Central Michigan University School of Medicine Clinical Institute, Children's Research Institute, Children's Hospital of Michigan, Detroit, Michigan; and* [3]*Department of Pediatrics, Wayne State University Clinical Research Service Center, Children's Hospital of Michigan, Detroit, Michigan*

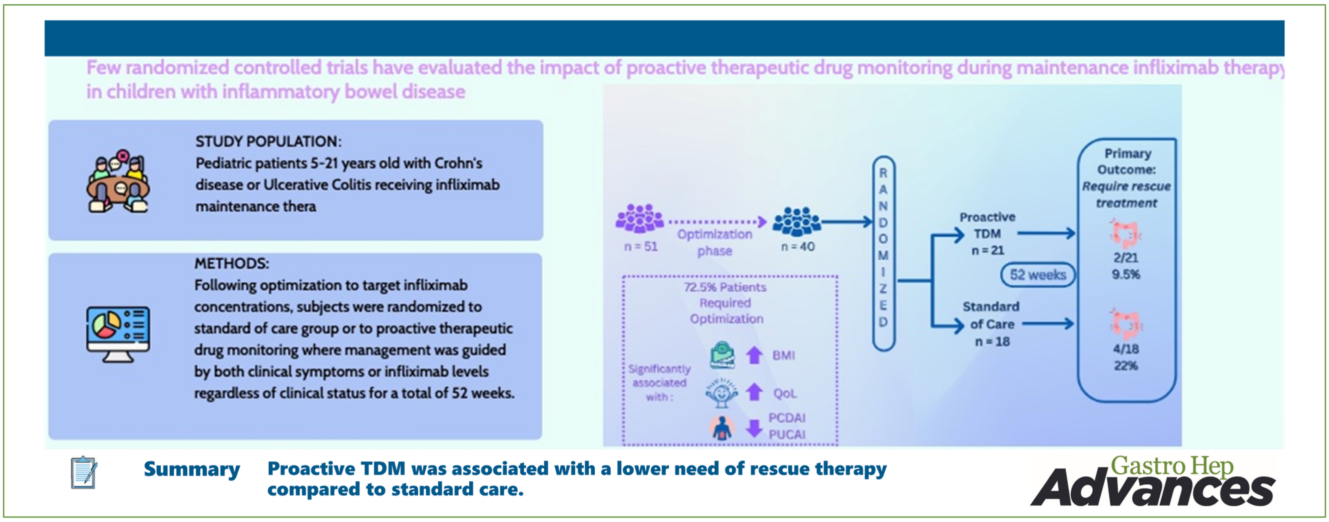

BACKGROUND AND AIMS: Despite potential benefits of anti-TNF proactive therapeutic drug monitoring (pTDM), few randomized controlled trials have assessed the impact of maintenance therapy in children with inflammatory bowel disease. **METHODS:** This single-center randomized controlled trial assessed pTDM in pediatric patients receiving infliximab maintenance therapy. Patients aged 5–21 years, diagnosed with moderate to severe Crohn's disease or ulcerative colitis, were enrolled. Following optimization to target infliximab concentrations, participants were randomized to either the standard of care (SOC) group, with treatment adjustments based on clinical presentation alone, or to pTDM, where management additionally incorporated infliximab levels regardless of clinical status. Clinical outcomes were monitored over 52 weeks. All authors had access to the study data and reviewed and approved the final manuscript. **RESULTS:** Fifty-one patients enrolled in the study, and 39 completed the trial; 72.5% required dose optimization prior to randomization, improving Pediatric Ulcerative Colitis Activity Index/Pediatric Crohn's Disease Activity Index scores from 9.13 to 6.31(P = .002). The primary outcome, need for rescue therapy, occurred more often in SOC than pTDM (22% vs 9.5%, P = .387). **CONCLUSION:** pTDM was associated with reduced need for rescue therapy compared to SOC. Although not statistically significant, these findings are clinically relevant, supporting potential benefits of pTDM in pediatric inflammatory bowel disease patients on infliximab maintenance therapy. Further investigation is required to refine the timing and optimal strategies for implementation.

Keywords: Inflammatory Bowel Disease; Pediatrics; Infliximab; Therapeutic Drug Monitoring

Abbreviations used in this paper: **ADA, antidrug antibody; CD, Crohn's disease; IBD, inflammatory bowel disease; LOR, loss of response; PCDAI, Pediatric Crohn's Disease Activity Index; PK, pharmacokinetic; pTDM, proactive therapeutic drug monitoring; PUCAI, Pediatric Ulcerative Colitis Activity Index; QOL, quality of life; RCT, randomized controlled trial; SOC, standard of care; UC, ulcerative colitis.**

Most current article

Published by Elsevier Inc. on behalf of American Gastroenterological Association Institute. This is an open access article under the CC BY license (http://creativecommons.org/licenses/by/4.0/).
2772-5723
https://doi.org/10.1016/j.gastha.2026.100953

Introduction

Infliximab is an IgG1 mouse-human chimeric monoclonal antibody that targets TNF, one of the key proinflammatory cytokines involved in inflammatory bowel disease (IBD). It was among the first biologics approved for IBD and has significantly transformed management due to its robust efficacy. Despite general success in treating IBD, 10%–40% of patients fail to respond to anti-TNF agents, termed primary nonresponse, while 30%–50% of patients who initially respond to treatment cease responding over time, termed secondary loss of response (LOR).[1–3] Primary nonresponse is likely due to a different key driver of inflammation other than TNF in these patients.[4] Secondary LOR occurs in patients who initially responded to anti-TNF therapy. The mechanisms behind secondary LOR are not fully understood, but are thought to involve either immune sensitization, due to the immunogenicity of infliximab, which can lead to the formation of antidrug antibodies (ADAs) or suboptimal drug levels due to rapid drug clearance.[1,5–7] Rapid drug clearance can be influenced by factors such as disease burden or patient's clinical characteristics.[8] For example, severe inflammatory burden can lead to high TNF antigen levels and rapid clearance through the upregulated reticuloendothelial system, in addition to leakage through inflamed bowel mucosa and low serum albumin. Other factors such as excessive adipose tissue or mesenteric fat can also increase TNF antigen load.[1,9] This rapid drug clearance lowers infliximab trough concentrations, increasing the likelihood of anti-TNF antibody production and ultimately leading to treatment failure.[10]

Therapeutic drug monitoring (TDM) has revolutionized medical decision-making by providing a more individualized approach, especially for infliximab therapy, due to its degree of variability amongst patients.[11] Reactive TDM involves measuring drug and ADA levels when patients show signs or symptoms of inflammation, with treatment adjustments made accordingly. Given the numerous studies showing that higher anti-TNF concentrations correlate with better therapeutic outcomes, reactive TDM for anti-TNF therapy is now highly recommended by multiple gastroenterology societies.[10,12–14] In contrast, proactive therapeutic drug monitoring (pTDM) involves scheduled drug level measurements after induction and during maintenance, regardless of disease activity, with the goal of maintaining adequate drug concentrations. Theoretically, pTDM allows clinicians to prevent LOR before a patient shows clinical signs of relapse or develops high antibody titers, which could necessitate switching to a different treatment.[15,16] However, this approach has not been endorsed by professional societies, as there is limited evidence supporting its benefits and lack of agreement among meta-analysis of high-quality adult studies.[12,17] That said, other studies suggest that routinely measuring induction TDM, infliximab levels at week 14 or postinduction, is associated with sustained durability.[2,18] This is particularly crucial in the pediatric population, as IBD tends to be more aggressive in children, impacting growth, bone development, and puberty.[1,10] In fact, the updated European Crohn's and Colitis Organisation-European Society for Paediatric Gastroenterology, Hepatology, and Nutrition guidelines for the medical management of pediatric Crohn's disease (CD) now recommend pTDM postinduction, or prior to fourth infusion.[19] Given the limited FDA-approved treatment options for pediatric IBD, pTDM during ongoing therapy may be especially beneficial in this population. In fact, current trends in clinical practice are increasingly favoring pTDM, particularly in pediatric patients.

To date, a handful of randomized controlled trials (RCTs) have evaluated outcomes of pTDM exclusively in patients receiving anti-TNF therapy for IBD, and the majority only included adults.[12] The Trough Concentration Adapted Infliximab Treatment (TAXIT) study was the first RCT to assess the efficacy of pTDM during maintenance therapy following drug optimization in adults with CD.[20] Although the primary outcomes did not reach statistical significance, secondary outcomes, such as durable remission and relapse rates, showed statistically significant differences between the pTDM vs and standard of care (SOC) arms.[20] However, adult studies may not be representative of children, who are growing and developing over time. In fact, the first pediatric RCT comparing proactive vs reactive TDM on maintenance therapy, Pediatric Crohn's Disease Adalimumab Level-based Optimization Treatment [PAILOT] trial, demonstrated significantly higher rates of corticosteroid-free clinical remission in patients proactively monitored.[21]

Despite these findings, the role of pTDM during maintenance infliximab therapy in children, particularly after dose optimization, remains insufficiently define. Guided by the TAXIT trial, the present study prospectively evaluated clinical outcomes in pediatric patients with IBD on infliximab maintenance therapy who were randomized to pTDM vs SOC following dose optimization for ongoing treatment management.

Methods

Study Design

This was a single center RCT assessing the benefits of proactive drug monitoring in patients with IBD receiving maintenance infliximab therapy. The study was conducted at the Children's Hospital of Michigan from May 2021 to June 2024 and was approved by the institutional review board (ClinicalTrials.gov ID NCT04921670).

Participants

Patients aged 5–21 years with moderate to severe CD or ulcerative colitis (UC) were eligible. Inclusion criteria required patients completing induction and in stable clinical status, as determined by both the treating physician and the primary investigator. Exclusion criteria included patients not receiving

maintenance infliximab therapy and those with an infliximab antibody titer greater than 1000 ng/mL or nonresponse to induction therapy. Additional exclusions were made during optimization phase for patients unable to complete the study due to persistently high antibody titers and those deemed at risk for noncompliance by the investigator. This was a pragmatic study, so varying infliximab doses were allowed. However, patients receiving doses above 12.5 mg/kg every 4 weeks prior to enrollment were excluded. Concomitant medications such as 5-aminosalicylic acid, azathioprine, methotrexate, budesonide, and stable, low-dose oral corticosteroids (defined as $\leq$0.5 mg/kg or $<$20 mg for patients over 40 kg) were permitted.

Interventions

This was a randomized clinical trial consisting of the following 2 phases: optimization and maintenance.

Optimization Phase

Infliximab dosing was adjusted for all patients using electrochemiluminescence immunoassays (LabCorp), targeting troughs between 5 and 20 ug/mL and little to no antibodies. The assay detects ADA in the presence of infliximab with detection thresholds categorized as low titer (22–200 ng/mL), moderate titer (201–1000 ng/mL), and high titer ($>$1000 ng/mL). If levels were suboptimal, dosing was intensified (increased by 2.5 mg/kg or shortened by 2 weeks) per the discretion of the treating physician. Immunomodulators such as azathioprine and/or methotrexate were permitted. Once target infliximab levels and minimal to no antibodies were achieved, patients advanced to the maintenance phase.

Maintenance Phase

Patients were randomized 1:1 to SOC or pTDM groups, with randomization managed using a computer-generated schedule by research team members not involved in clinical care. Infliximab assays were obtained for all participants prior to each infusion. The SOC group followed standard clinical practice, with dosing adjustments based on clinical disease activity and/or standard laboratory values (complete blood count, C-reactive protein, sedimentation rate, and albumin). The pTDM group was managed based on infliximab trough concentrations and ADA results as well as clinical disease activity and standard laboratory markers. Infliximab assays were obtained at every infusion for both the SOC and pTDM groups, but treatment adjustments based on the infliximab assays alone were performed on the pTDM groups only. Dose adjustments mirrored the optimization phase. Addition of immunomodulators was allowed.

The primary gastroenterologists were permitted to adjust infliximab dosing during both optimization and maintenance phases as per SOC, but they were unaware of the assay results and unable to manage based upon those results. Clinical disease activity and quality of life (QOL) were assessed at every infusion using the Pediatric Crohn's Disease Activity Index (PCDAI), Pediatric Ulcerative Colitis Activity Index (PUCAI), and EQ-5D instruments (https://euroqol.org/eq-5d-instruments/).[22] Participants completed the study 52 weeks from randomization. Research Electronic Data Capture system was used for data collection.

Outcomes

The primary outcome was to compare the number of patients requiring rescue treatment (eg, steroids, additional immunomodulators, or increased infliximab) during the maintenance phase, 1 year postoptimization. Secondary outcomes included clinical and biochemical remission before/after optimization and at the study end. Sustained remission (every infusion) was tracked. Clinical remission was defined as PUCAI or PCDAI scores $<$10, and biochemical remission was defined as CRP $<$5 mg/L. Other inflammatory markers (ESR, albumin) were recorded but were not used to define remission. Additional outcomes included comparing the proportion of patients with ADA levels and QOL changes at the end of maintenance phase.

Statistical Analysis

Parametric independent samples *t*-tests compared mean relapses. Nonparametric Mann-Whitney *U* tests were used for skewed data. Fisher exact test compared categorical variables; *t*-tests or Mann-Whitney *U* tests were used for continuous variables. SPSS (version 28.0) was used for analysis (IBM Inc, Chicago, IL).

Results

Patient Population

Fifty-one patients were enrolled (median age: 12 years; interquartile range: 7–17; 42% were female). Thirty-seven (72%) were diagnosed with CD and 14 (27%) with UC.

Optimization Phase

Eleven patients were excluded during optimization (Figure 1), primarily due to elevated ADAs (27%). Forty patients completed the optimization phase, with 29 (72.5%) requiring dose optimization (26 had dose adjustments, 1 added an immunomodulator, and 2 required both) (Supplementary Table 1).

Patients who completed the optimization phase were included in preoptimization and postoptimization comparisons. Anthropometric measures showed significant improvements, with weight increasing from 63.5 $\pm$ 24 kg to 66.16 $\pm$ 24.49 kg ($P = .006$) and body mass index from 23.11 $\pm$ 8.26 kg/m^2 to 24.9 $\pm$ 7.47 kg/m^2 ($P = .026$) (Supplementary Table 2). Biochemical indices, including hemoglobin, albumin, or CRP, remained stable, with values either mildly abnormal or normal for both preoptimization and postoptimization. PCDAI and PUCAI significantly improved from 9.13 $\pm$ 9.53 to 6.31 $\pm$ 6.88 ($P = .002$), with clinical remission increasing from 52.5% to 70%. Infliximab dose increased from 7.81 $\pm$ 2.38 mg/kg to 9.24 $\pm$ 2.06 mg/kg ($P = .001$), and frequency decreased from 7.63 to 6.9 weeks ($P \leq .001$). Infliximab trough levels rose from 6.04 $\pm$ 3.82 ug/dL to 9.31 $\pm$ 3.58 ug/dL ($P \leq .001$), and the proportion of patients with abnormal troughs decreased from 60% to 35% ($P = .043$). QOL scores—with 5 being best health and 25 being worst—improved, with patient

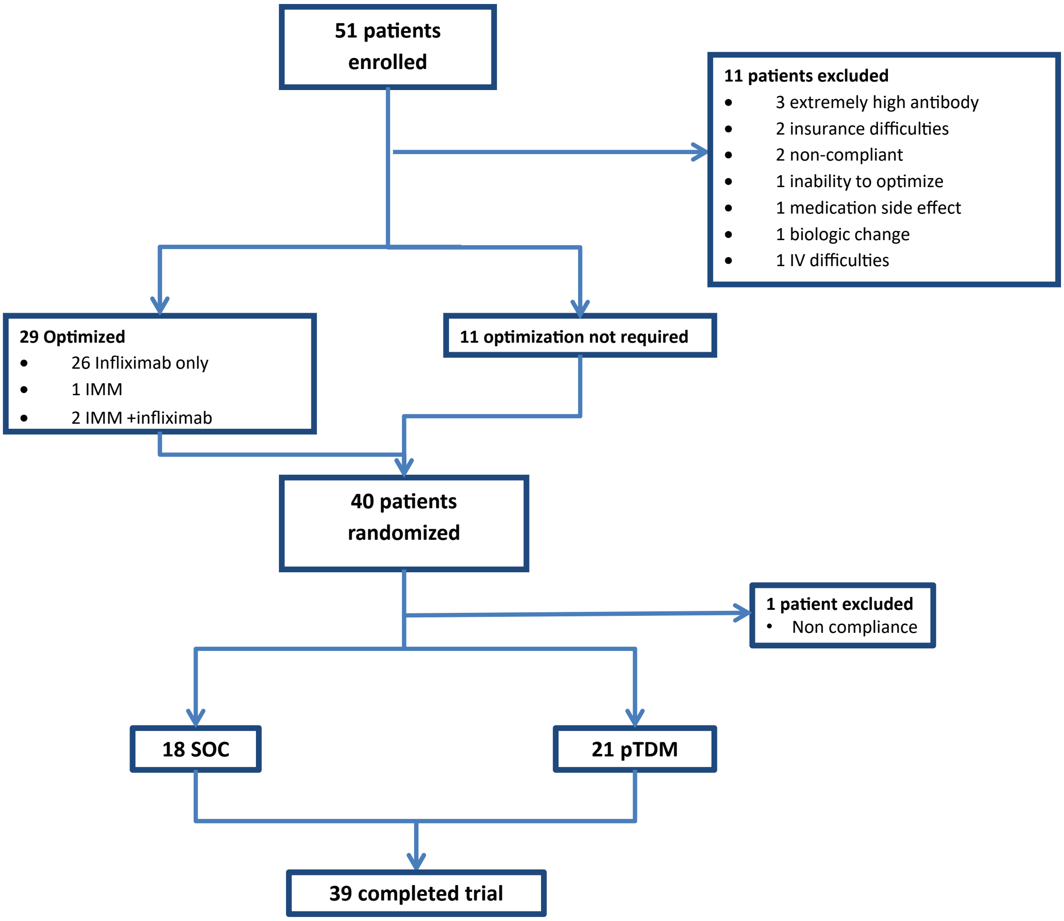

Figure 1. Flowchart detailing study population. IMM, immunomodulator; pTDM, proactive therapeutic drug monitoring; SOC, standard of care.

scores decreasing from 6.31 ± 1.76 to 5.92 ± 1.09 (P = .096), and parents' scores from 6.69 ± 2.21 to 6.00 ± 1.44 (P = .017).

Maintenance Phase

After randomization, 1 participant was excluded due to noncompliance leaving 39 patients who completed the trial (18 SOC, 21 pTDM). Of these, 15 (83%) in the SOC group and 15 (71%) in the pTDM group were diagnosed with CD, and 3 (17%) and 6 (29%) with UC in SOC and pTDM group, respectively (P = .38). Among CD patients, the majority had an inflammatory phenotype (86%) in the SOC group, followed by penetrating (7%) and stricturing (7%); in the pTDM group, 79% had an inflammatory phenotype, while 7% had penetrating, stricturing, or both penetrating and stricturing (P = .79). Three CD patients (20%) in the SOC had perianal involvement, compared to 6 (40%) in pTDM group (P = .23). Among UC, the extent of involvement was primarily pancolonic for both SOC (3 patients, 100%) and pTDM (3 patients, 50%). Rectal involvement was observed in 1 patient (17%) and left-sided disease in 2 patients (33%) in the pTDM group only (P = .32). Mean disease duration prior to enrollment was 43 ± 34 months in the SOC group and 33 ± 31 months in pTDM group (P = .36). Mean duration of infliximab use prior to enrollment was 30 ± 32 months for SOC group, 23 ± 32 months in the pTDM group (P = .45). Three patients (17%) in the SOC group and 8 patients (38%) in the pTDM group were on concomitant therapy prior to randomization (P = .14). One patient (6%) in the SOC group and 4 patients (19%) in the pTDM group had a history of IBD-related surgery (P = .21). Three patients (17%) had comorbidities in SOC group, 6 patients (29%) in pTDM group (P = .38). (Table 1).

Primary Outcome

Rescue therapy was required in 4 SOC (22%) vs 2 pTDM (9.5%) patients (P = .387) (Figure 2). Of the 4 SOC, 3 underwent infliximab dose escalation, while one required both infliximab optimization and steroid use. Infliximab dose escalation alone was required in the pTDM patients (Supplementary Table 1).

Secondary Outcome

There were no significant differences between groups in clinical remission (defined as PUCAI or PCDAI <10), with 14 of 18 (77.8%) in the SOC group and 14 of 21 (66.7%) in

Table 1. Demographics/Characteristics

Demographics/characteristics	SOC (n = 18)	pTDM (n = 21)	*P*
Age at diagnosis, (y), mean ± SD	10 (3)	12 (3)	.17
Sex, female, n (%)			
Race (n)			.36
African American	7 (39)	6 (29)	
Middle Eastern	0	1 (5)	
White	6 (33)	11 (52)	
African American ± Caucasian	2 (11)	1 (5)	
Other	3 (17)	2 (9)	
Duration of disease (mo), mean ± SD	43 (34)	33 (31)	.36
Duration on infliximab (mo), mean ± SD	30 (32)	23 (32)	.45
Diagnosis, n (%)			.38
Crohn's disease	15 (83)	15 (71)	
Ulcerative colitis	3 (17)	6 (29)	
Crohn's phenotype, n (%)			.79
Inflammatory	13 (86)	12 (79)	
Stricturing	1 (7)	1 (7)	
Penetrating	1 (7)	1 (7)	
Penetrating + stricturing	0	1 (7)	
Perianal disease, n (%)	3 (20)	6 (40)	.23
Ulcerative colitis extent, n (%)			.32
Rectal	0	1 (17)	
Left sided	0	2 (33)	
Extensive	0	0	
Pancolonic	3 (100)	3 (50)	
Concomitant medications, n (%)	3 (17)	8 (38)	.14
Azathioprine	1 (33)	2 (25)	
Methotrexate	1 (33)	4 (50)	
5-ASA	0	2 (25)	
Methotrexate +5-ASA	1 (33)	0	
Surgical history, n (%)	1 (6)	4 (19)	.21
Perianal incision and drainage	1 (100)	1 (25)	
Perianal granulation tissue excision	0	1 (25)	
Ileocecectomy	0	1 (25)	
Intra-abdominal abscess drainage and ileocecectomy	0	1 (25)	
Comorbidities, n (%)	3 (17)	6 (29)	.38
Iron deficiency anemia	1 (33)	4 (66)	
Arthritis	1 (33)	1 (17)	
Psoriasis	0	1 (17)	
Pyoderma gangrenosum	1 (33)	0	

ASA, aminosalicyclic-acid; IQR, interquartile range; SD, standard deviation; SOC, standard of care; TDM, proactive therapeutic drug monitoring.

the pTDM group (P = .342) (Figure 3). Additionally, steroid-free clinical remission was similar in both groups with 13 patients in both SOC (72%) and pTDM (62%) groups (P = .5). The average PCDAI/PUCAI score was 8.75 ± 18.01 in the SOC group vs 7.26 ± 9.25 in the pTDM group (P = .371). Disease outcomes in regards to surgeries, hospitalizations, and infusion reactions did not differ between the 2 groups. Biochemical remission, including hemoglobin, albumin, CRP, and ESR, was also similar between both groups (Table 2). See Figure 4 for a survival analysis of time to rescue therapy. Additionally, there were no significant differences between groups when comparing CD vs UC in terms of rescue therapy (20% in SOC and 7% in pTDM for CD, P = .28; 33% in SOC and 17% in pTDM for UC, P = .57) or clinical remission (87% in SOC and 80% in pTDM for CD, P = .62; 33% in both SOC and pTDM for UC, P = 1.

Infliximab Pharmacokinetics

There were no statistically significant differences in infliximab dosing between groups (SOC: 9.28 ± 2.36 mg/kg vs pTDM: 9.67 ± 1.65 mg/kg). In the SOC group, 8 out of 18 (44.4%) patients had abnormal infliximab assays, with low trough levels in 4 out of 18 (22.2%) and 6 out of 18 (33.3%) with elevated antibody levels. In the pTDM group, 7 out of 20 (35%) had abnormal assays, with 3 out of 20 (15%) showing low trough levels and 6 out of 21 (28.6%) with elevated antibodies. The mean infliximab concentration was 8.51 ± 4.39 ug/dL in the SOC group and 9.51

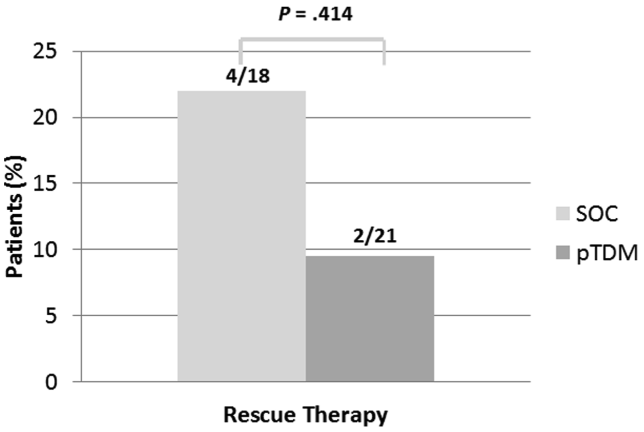

Figure 2. Primary end point: percentage requiring rescue therapy (defined as need for steroids, additional immunomodulator, or increased IFX dose) between standard of care and therapeutic drug monitoring arm. pTDM, proactive therapeutic drug monitoring; SOC, standard of care.

± 4.73 ug/dL in the pTDM group (P = .497). Mean infliximab antibody levels were higher in the SOC group 41.06 ± 36.54 ng/mL compared to 26.9 ± 15.99 ng/mL in the pTDM group (P = .117) (Table 2). For the patients who required rescue therapy, the mean infliximab level prior to randomization was 6 ug/dL compared to 10 ug/dL for those who did not require rescue therapy (P < .001). A Mann-Whitney U Test, comparing the postmaintenance level of abnormal antibody titers between SOC and pTDM, revealed the majority of abnormal ATI levels were lower in the pTDM compared to the SOC group (Figure 5).

Quality of Life

Patient and parent QOL scores were similar between both groups. Patient mean survey scores, with 5 indicating the best overall health and 25 being worst, were 6.28 ± 2.19 in SOC group vs 6.14 ± 1.74 in the pTDM group (P = .832). Parent mean scores were slightly higher in the pTDM group (5.71 ± 1.44) compared to SOC (6.4 ± 2.06 P = .312). The sliding scale of overall health (0–100, with 100 being the best health) also showed similar results between groups (Table 2).

Adverse Events

A total of 12 adverse events were reported, with 1 potentially related to infliximab (psoriasis improved after discontinuation). Five events were of unknown relationship to infliximab. There were no serious adverse events (Supplementary Table 3).

Discussion

In this randomized trial comparing pTDM to SOC, patients in the pTDM group were more likely to undergo dose escalation, achieve higher infliximab trough levels, and

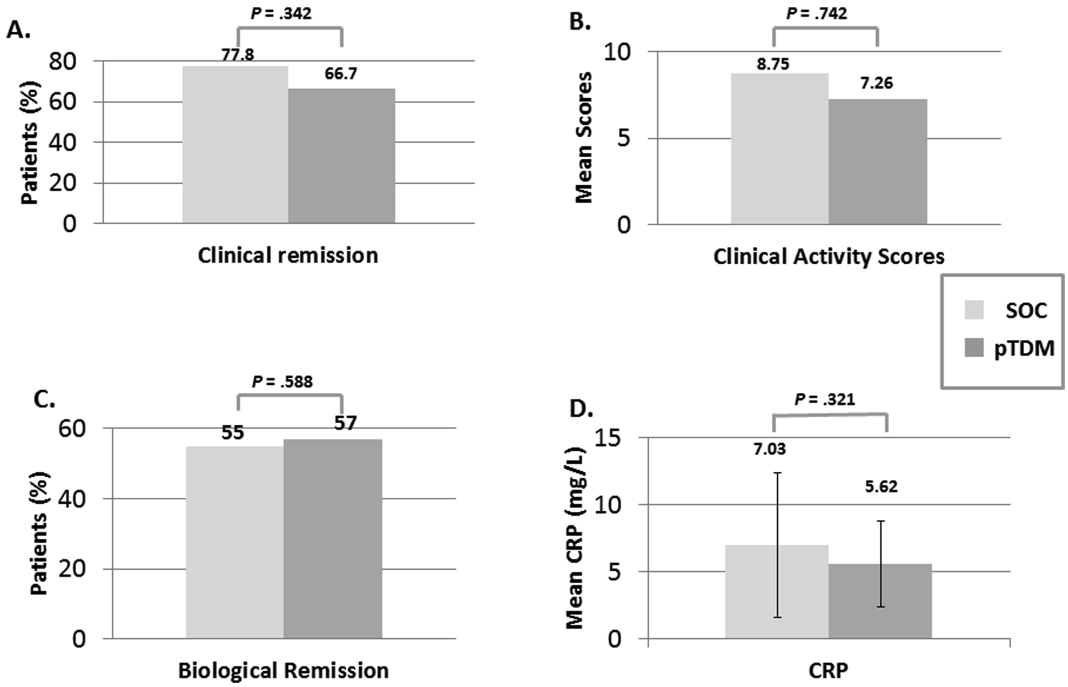

Figure 3. Secondary end-points. (A) Proportion of subjects in clinical remission at the end of maintenance phase, defined as PUCAI and PCDAI scores <5. (B) Mean PUCAI and PCDAI scores between SOC and TDM at the end of maintenance phase. (C) Proportion of patients in biologic remission, defined as CRP <5 mg/L. (D) Mean CRP values between SOC and TDM at the end of maintenance phase. pTDM, proactive therapeutic drug monitoring; SOC, standard of care.

Table 2. End of Maintenance Phase Characteristics of SOC vs TDM Group

Characteristics	SOC	TDM	Mean diff/Std error	95% CI	*P*
Weight (kg)	67.25 ± 26.65	67.81 ± 22.43	0.55 ± 7.86	−0.65 to 0.60	.944
Weight Z score	0.69 ± 1.29	0.69 ± 1.11	−0.00 to 0.41	−0.83 to 0.82	.990
Height (cm)	162.05 ± 10.91	163.38 ± 13.94	1.33 ± 4.17	−9.78 to 7.14	.752
Height Z score	9.19 ± 37.85	−0.13 ± 1.07	9.32 ± 8.67	−8.31 to 26.9	.291
BMI (kg/m^2)	26.32 ± 10.48	24.48 ± 5.96	1.85 ± 2.68	−3.59 to 7.28	.496
BMI Z score	0.75 ± 1.38	0.67 ± 1.16	0.08 ± 0.43	−0.79 to 0.96	.850
Hemoglobin (g/dL)	12.86 ± 1.32	13.23 ± 1.82	0.37 ± 0.52	−1.41 to 0.67	.476
Albumin (g/dL)	4.24 ± 0.40	4.25 ± 0.22	0.01 ± 0.10	−0.21 to 0.20	.938
C-reactive protein (mg/L)	7.03 ± 5.39	5.62 ± 3.19	1.41 ± 1.37	−1.42 to 4.23	.321
Erythrocyte sedation rate (mm/h)	14.28 ± 10.77	9.76 ± 8.53	4.52 ± 3.09	−1.75 to 10.8	.152
PCDAI	4.50 ± 7.74	4.17 ± 5.72	0.33 ± 2.49	−4.78 to 5.45	.894
PUCAI	30.00 ± 39.05	15.00 ± 12.25	15.00 ± 16.48	−23.96 to 53.96	.393
PCDAI/PUCAI	8.75 ± 18.01	7.26 ± 9.25	1.48 ± 4.49	−7.6 to 10.58	.371
Clinical remission, n (%)	14/18 (77.8)	14/21 (66.7)			.342
Treatment med optimization, n (%)	4/18 (22.2)	5/21 (23.8)			.605
Number of optimizations per patient, n	1.85 ± .14	2.08 ± 1.08	0.24 ± 0.45	−1.16 to 0.68	.600
Optimization due to disease, n (%)	4/18 (22)	2/21 (9.5)			0.387
Dose infliximab (mg/kg)	9.28 ± 2.36	9.67 ± 1.65	0.39 ± 0.65	−1.70 to 0.91	.545
Frequency of infliximab (wk)	7.00 ± 1.03	6.76 ± 1.00	0.24 ± 0.33	−0.42 to 0.89	.468
Abnormal infliximab assay, n (%)	8/18<br>44.4%	7/20<br>38.0%			.396
Infliximab trough concentration (ug/dL)	8.51 ± 4.39	9.51 ± 4.73	−1.01 ± 1.47	−3.99 to 1.97	.497
Abnormal infliximab trough, n (%)	4/18<br>22.2%	3/20<br>15.0%			.437
Infliximab antibody (ng/mL)	41.06 ± 36.54	26.90 ± 15.99	14.15 ± 8.81	−3.69 to 31.99	.117
Abnormal infliximab antibody, n (%)	6/18<br>33.3%	6/21<br>28.6%			.509
Patient QOL survey (scale 5–25, 5 = best health)	6.28 ± 2.19	6.14 ± 1.74	.14 ± .63	−1.14 to 1.41	.832
Patient well-being scaling score (100 = best health)	89.78 ± 15.01	89.76 ± 12.92	0.02 ± 4.47	−9.04 to 9.07	.997
Parent reported QOL survey (scale 5–25, 5 = best health)	5.71 ± 1.44	6.40 ± 2.06	0.69 ± 0.67	−2.05 to 0.68	.312
Parent well-being scaling score (100 = best health)	92.85 ± 7.32	89.07 ± 13.52	3.78 ± 4.21	−4.87 to 12.43	.377

BMI, body mass index; CI, confidence interval.

exhibit lower antibody titers. Additionally, a higher proportion of patients in the SOC group required rescue therapy compared to the pTDM group (22% vs 9.5%), a clinically noteworthy finding despite the lack of statistical significance in the context of a small sample size. These results suggest that pTDM may provide additional benefit even in children already optimized on maintenance therapy.

Several RCT have examined the impact of anti-TNF proactive drug monitoring, but the results have been inconsistent.[12,17,23] Nguyen et al[12] conducted a systematic review and meta-analysis of all 9 RCTs, primarily in adults, and found no benefit of proactive over reactive monitoring in maintaining clinical remission. However, only 1 study included children, the results from which were overshadowed by adult studies. Similarly, a more recent meta-analysis by Marcos et al,[23] which focused on the highest-quality RCTs, also found no clear advantage of pTDM. Both reviewed highlighted heterogeneity and limited number of studies as key factors contributing to the lack of consensus. Based on these findings, we chose to adopt a methodology similar to the TAXIT study, the first RCT on exclusive infliximab proactive vs reactive TDM, in order to ensure consistency and comparability with a previous high-quality trial.

Studies continue to indicate that various factors influence the pharmacokinetics (PKs) of anti-TNF, particularly in children.[24] Pediatric patients, who often present with more extensive or aggressive disease, are more susceptible to increased drug clearance due to factors like hypoalbuminemia, lower body weight, higher TNF burden, and an overactive reticuloendothelial system.[1,10,24–27] Extrapolating PK data from adult studies is problematic, which underscores the need for further research on the response to anti-TNF in children.[26] Furthermore, unlike the meta-analyses by Nguyen and Marcos, which involved mostly adult patients, several prospective and retrospective pediatric studies have demonstrated the benefits of proactive drug monitoring.[2,10,16–18,25,28–32] Consequently, a key

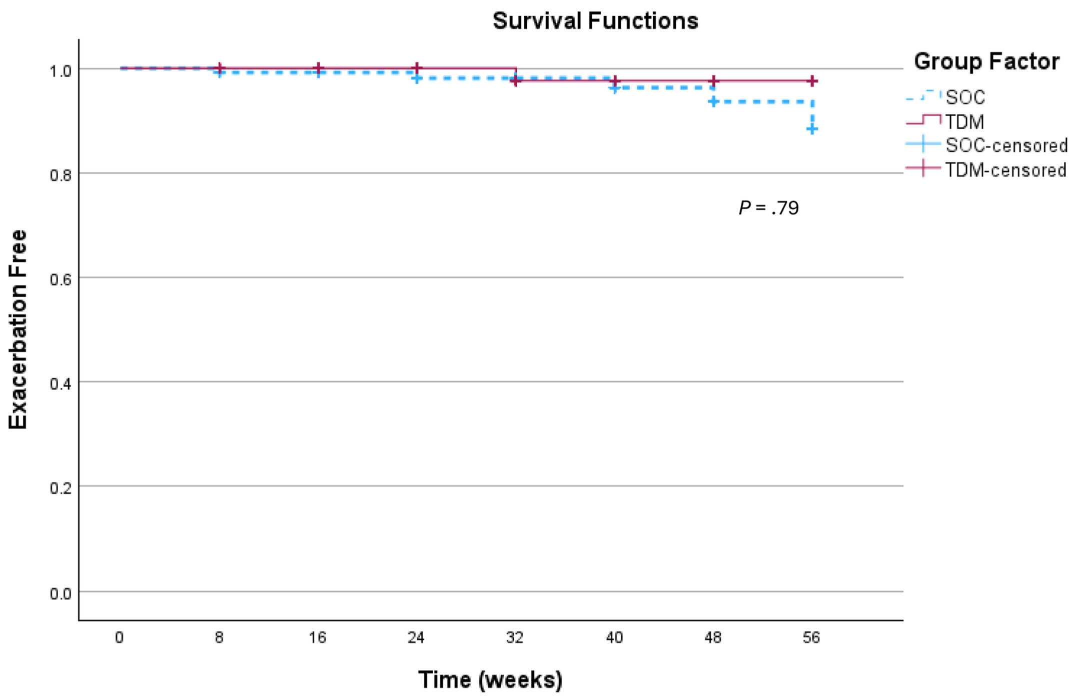

Figure 4. Survival analysis of time to the need of rescue therapy.

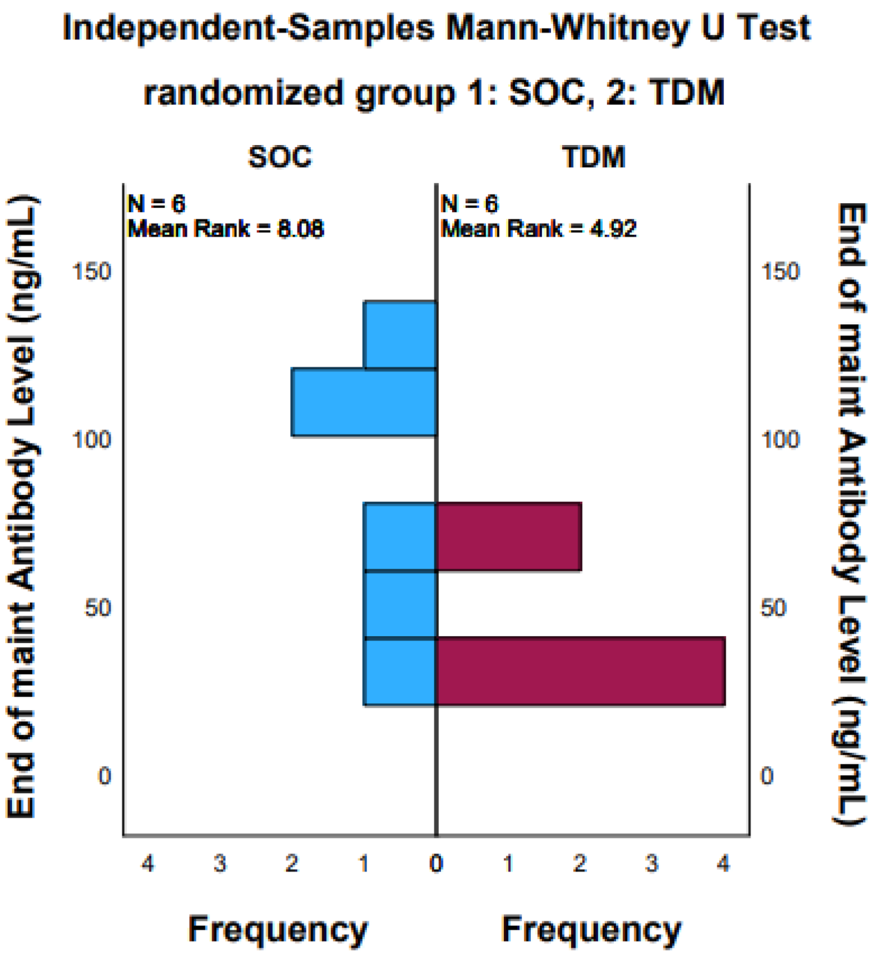

Figure 5. Mann-Whitney *U* test comparing abnormal antibody levels between SOC and TDM group post-maintenance phase. SOC, standard of care; TDM, therapeutic drug monitoring.

objective of our trial was to assess whether pediatric patients respond differently to pTDM during maintenance therapy.

The PAILOT trial was the first RCT specifically comparing the effect of dose adjustments to anti-TNF maintenance therapy based on proactive vs reactive drug monitoring in pediatric IBD.[21] In this trial, anti-TNF naïve children with CD receiving adalimumab were randomized to proactive vs reactive drug monitoring before the third injection and monitored for 72 weeks.[21] Patients in the proactive drug monitoring group achieved higher rates of clinical and biochemical remission, both throughout the trial and its conclusion. Notably, 90% required dose escalation to maintain therapeutic drug levels. Kang et al[33] recently published results of the second RCT comparing infliximab pTDM to reactive drug monitoring in pediatric CD, examining not only clinical remission but also endoscopic healing. Similarly, patients randomized to pTDM demonstrated higher rates of both endoscopic and steroid-free clinical remission by the end of 54 weeks. These studies highlight the need for more intensive drug monitoring in pediatric patients compared to adults and has been supported by subsequent studies.[24,27] In fact, a recent position paper written by the North American Society for Pediatric Gastroenterology, Hepatology, and Nutrition IBD Committee recommended consideration of proactive infliximab monitoring every 6–12 months during maintenance while noting that optimal timing remains uncertain.[34]

Remarkably, 72.5% of participants required dose adjustments during optimization. Statistically significant improvements in clinical disease activity, anthropometric measurements, and QOL scores were observed post-optimization. It is also important to note that 3 patients were removed from the study due to extremely abnormal infliximab assays detected at enrollment—findings that would have otherwise gone undetected. Comparisons were limited to those completing optimization, potentially underestimating the overall impact. These results reinforce the value of pTDM. Additionally, despite entering randomization with therapeutic drug levels for each group, although not statistically significant, patients in the pTDM arm required less rescue therapy and had lower antibody titers compared to SOC, as seen in Figure 5. This also suggests that ongoing proactive monitoring may offer additional benefits. However, the optimal timing of continued pTDM remains an unanswered question.

Advances in PK modeling are transforming personalized anti-TNF management.[24–27,35–39] PK dashboards, such as RoadMAB and iDOSE, integrate clinical, biochemical, and drug-level data to predict infliximab clearance and optimize dosing.[25,38] Early studies demonstrate improved trough level maintenance, reduced LOR, and enhanced disease control in both induction and maintenance settings. Trials such as REMODEL and OPTIMIZE are currently evaluating these tools in pediatric populations, while adult studies, like PRECISION, confirm the feasibility and benefit of PK-guided dosing.[24,38] Together, these approaches represent a new era of individualized care, particularly relevant in children with high interpatient variability.

Several limitations must be acknowledged. First, the small sample size limited statistical power to detect significant differences between groups. Second, insurance denials forced transitions to external infusion centers, reducing eligibility and retention, as the study design required infusions at a single site with a standardized infliximab assay to reduce variability. These transitions only occurred during the enrollment or optimization phases and did not affect patients following randomization.

Third, the 1-year study duration may have been insufficient to fully capture impact of pTDM, given the number of patients who required dose adjustments required during optimization. Additionally, disease activity was assessed using PCDAI and PUCAI rather than fecal calprotectin or endoscopy, tests which may have provided a more comprehensive assessment of disease activity. Lastly, we acknowledge that the study population consisted of patients already on maintenance infliximab who had undergone dose optimization prior to randomization. As a result, most patients entered randomization with therapeutic drug levels. Despite this, the pTDM group demonstrated fewer patients requiring rescue therapy and lower ADA titers, suggesting that pTDM within 1 year may offer additional benefit, even in those with initially optimized therapy. Overall, this study suggests that pTDM is helpful; however, how a provider utilizes pTDM, specifically its timing, was outside the scope of this study and remains an unresolved question.

Conclusion

As personalized approaches to anti-TNF dosing continues to evolve, our findings support the clinical utility of pTDM during maintenance therapy, particularly in identifying and addressing issues that may go unnoticed in routine clinical care. However, optimal timing and implementation strategies remain areas for further study, especially given the unique PK considerations in pediatric patients.

Supplementary Materials

Material associated with this article can be found, in the online version, at https://doi:10.1016/j.gastha.2026.100953.

References

1. Carman N, Mack DR, Benchimol EI. Therapeutic drug monitoring in pediatric inflammatory bowel disease. Curr Gastroenterol Rep 2018;20(5):18.
2. Lega S, Phan BL, Rosenthal CJ, et al. Proactively optimized infliximab monotherapy is as effective as

combination therapy in IBD. Inflamm Bowel Dis 2019;25(1):134–141.
3. Dubinsky MC, Mendiolaza ML, Phan BL, et al. Dashboard-driven accelerated infliximab induction dosing increases infliximab durability and reduces immunogenicity. Inflamm Bowel Dis 2022;28(9):1375–1385.
4. Rutgeerts PJ. Therapeutic monitoring of anti-TNF levels and antibodies to predict response and achieve mucosal healing. Gastroenterol Hepatol (N Y) 2014;10(7 Suppl 4):1–20.
5. Vande Casteele N, Feagan BG, Wolf DC, et al. Therapeutic drug monitoring of tumor necrosis factor antagonists in crohn disease: a theoretical construct to pharmacokinetics and guidelines to clinical practice. Inflamm Bowel Dis 2021;27(8):1346–1355.
6. Zitomersky N, Chi L, Liu E, et al. Anti-infliximab antibodies and low infliximab levels correlate with drug discontinuation in pediatric inflammatory bowel disease. J Pediatr Gastroenterol Nutr 2024;78(2):261–271.
7. Naviglio S, Lacorte D, Lucafo M, et al. Causes of treatment failure in children with inflammatory bowel disease treated with infliximab. J Pediatr Gastroenterol Nutr 2019;68(1):37–44.
8. Stallard L, Frost K, Frost N, et al. Body surfaced area based-dosing of infliximab is superior to standard weight-based dosing in children with very early onset inflammatory bowel disease. Gastro Hep Adv 2023;3(2):215–220.
9. Whaley KG, Xiong Ye, Karns R, et al. Multicenter cohort study of Infliximab pharmacokinetics and therapy response in pediatric acute severe ulcerative colitis. Clin Gastroenterol Hepatol 2023;21(5):1338–1347.
10. Papamichael K, Stocco G, Ruiz del Agua A. Challenges in therapeutic drug monitoring: optimizing biological treatments in patients with inflammatory bowel disease and other immune-mediated inflammatory diseases. Ther Drug Monit 2023;45(5):579–590.
11. Papamichael K, Jairath V, Zou G, et al. Proactive infliximab optimization using a pharmacokinetic dashboard versus standard of care in patients with Crohn's disease: study protocol for a randomised, controlled, multicentre open-label stud (the OPTIMIZE trial). BMJ Open 2022;12(4):e057656.
12. Nguyen NH, Solitano V, Vuyyuru SK, et al. Proactive therapeutic drug monitoring versus conventional management for inflammatory bowel diseases: a systematic review and meta-analysis. Gastroenterology 2022;163(4):937–949.e2.
13. Vaughn BP, Martinez-Vazquez M, Patwardhan V, et al. Prospective therapeutic drug monitoring and optimization of infliximab maintenance therapy in IBD. Gastroenterol Hepatol (N Y) 2014;10(7 Suppl 4):1–20.
14. Gasparetto M, Burgess N, Naik S, et al. Advantages of proactive therapeutic drug monitoring in a prospective cohort of children with inflammatory bowel disease treated with anti-TNF necrosis factor. J Pediatr Gastroenterol Nutr 2022;74(4):484–489.
15. Vaughn BP, Martinez-Vazquez M, Patwardhan VR, et al. Proactive therapeutic concentration monitoring of infliximab may improve outcomes for patients with inflammatory bowel disease: results from a pilot observational study. Inflamm Bowel Dis 2014;20(11):1996–2003.
16. Burgess CJ, Reilly C, Steward-Harrison L, et al. Utility of proactive infliximab levels in paediatric Crohn's disease. Arch Dis Child 2019;104(3):251–255.
17. Sethi S, Dias S, Kumar A, et al. Meta-analysis: the efficacy of therapeutic drug monitoring of anti-TNF-Therapy in inflammatory bowel disease. Aliment Pharmacol Ther 2023;57(12):1362–1374.
18. Singh N, Rosenthal CJ, Melmed GY, et al. Early infliximab trough levels are associated with persistent remission in pediatirc patients with inflammatory bowel disease. Inflamm Bowel Dis 2014;20(10):1708–1713.
19. Van Rheenen PF, Aloi M, Assa A, et al. The medical management of paediatric Crohn's disease: an ECCO-ESPGHAN guideline update. J Crohns Colitis 2020;7:jjaa161.
20. Vande Casteele N, Ferrante M, Van Assche G, et al. Trough concentrations of Infliximab guide dosing for patients with inflammatory bowel disease. Gastroenterology 2015;148(7):1320–1329.e3.
21. Assa A, Matar M, Turner D, et al. Proactive monitoring of adalimumab trough concentration associated with increased clinical remission in children with Crohn's disease compared with reactive monitoring. Gastroenterology 2019;157(4):985–996.e2.
22. Rabin R, de Charro F. EQ-5D: a measure of health status from the EuroQoL Group. Ann Med 2001;33(5):337–343.
23. Marcos NM, Arribas BN, Navarro GM, et al. Efficacy and safety of proactive drug monitoring in inflammatory bowel disease treated with anti-TNF agents: a systematic review and meta-analysis. Dig Liver Dis 2024;56(3):421–428.
24. Minar PP, Colman RJ, Zhang N, et al. Precise infliximab exposure and pharmacodynamic control to achieve deep remission in paediatric Crohn's disease (REMODEL-CD): study protocol for a multicentre, open-label, pragmatic clinical trial in the USA. BMJ Open 2024;14(3):e077193.
25. Colman RJ, Xiong Y, Mizuno T, et al. Antibodies-to-Infliximab clearance while dose intensification reverses immunogenicity and recaptures clinical response in paediatric Crohn's disease. Aliment Pharmacol Ther 2022;55(5):593–603.
26. Winter DA, Joosse ME, de Wildt SN, et al. Pharmacokinetics, pharmacodynamics, and immunogenicity of infliximab in pediatric inflammatory bowel disease: a systematic review and revised dosing considerations. J Pediatr Gastroenterol Nutr 2020;70(6):763–776.
27. Xiong Y, Mizuno T, Colman R, et al. Real-world infliximab pharmacokinetic study informs an electronic health record-embedded dashboard to guide precision dosing in children with Crohn's disease. Clin Pharmacol Ther 2021;109(6):1639–1647.
28. Lyles JL, Mulgund AA, Bauman LE, et al. Effect of a practice-wide anti-TNF proactive therapeutic monitoring program on outcomes in pediatric patients with inflammatory bowel disease. Inflamm Bowel Dis 2021;27(4):482–492.
29. Fernandes SR, Bernardo S, Sioes C, et al. Proactive Infliximab drug monitoring is superior to conventional management in inflammatory bowel disease. Inflamm Bowel Dis 2020;26(2):263–270.

30. Pinto Pais I, Espinheira MC, Trindade E, et al. Optimizing antitumor necrosis factor treatment in pediatric inflammatory bowel disease with therapeutic drug monitoring. J Pediatr Gastroenterol Nutr 2020;71(1):12–18.
31. Minar P, Saeed SA, Afreen M, et al. Practical use of Infliximab concentration monitoring in pediatric Crohn's disease. J Pediatr Gastroenterol Nutr 2016;62(5):715–722.
32. van Hoeve K, Dreesen E, Hoffman I. Higher infliximab trough levels are associated with better outcome in paediatric patients with inflammatory bowel disease. J Crohns Colitis 2018;12(11):1316–1325.
33. Kang B, Kim ES, Choi S, et al. Proactive drug monitoring versus clinically based dosing for endoscopic healing in pediatric Crohn's disease receiving infliximab. Clin Gastroenterol Hepatol 2026;24:201–209.e6.
34. Felipez LM, Ali S, de Zoeten EF, et al. North American Society for Pediatric Gastroenterology, Hepatology, and Nutrition position paper on the therapeutic drug monitoring in pediatric inflammatory bowel disease. J Pediatr Gastroenterol Nutr 2025;81:1100–1117.
35. Colman RJ, Samuels A, Mizuno T, et al. Model-informed precision dosing for biologics is now available at the bedside for patients with inflammatory bowel disease. Inflamm Bowel Dis 2023;29(8):1342–1346.
36. TITRATE (induction for acuTe ulcerative Colitis). Clinical Trials.gov Identifier: NCT03937609. Sponsor: Academisch Medisch Centrum – Universiteit van Amsterdam. https://clinicaltrials.gov/study/NCT03937609. Accessed March 7, 2025.
37. Proactive infliximab optimization using a pharmacokinetic dashboard versus standard of care in patients with inflammatory bowel disease: the OPTIMIZE trial. Clinical Trials.gov Identifier: NCT04835506. Sponsor: Beth Israel Deaconess Medical Center. https://clinicaltrials.gov/study/NCT04835506. Accessed March 7, 2025.
38. Strik AS, Lowenberg M, Mould DR, et al. Efficacy of dashboard driven dosing of infliximab in inflammatory bowel disease patients; a randomized controlled trial. Scand J Gastroenterol 2021;56(2):145–154.
39. Bauman LE, Xiong Y, Mizuno T, et al. Improved population pharmacokinetic model for predicting optimized infliximab exposure in pediatric inflammatory bowel disease. Inflamm Bowel Dis 2020;26(3):429–439.

Received December 9, 2025. Accepted March 31, 2026.

Correspondence:
Address correspondence to: Kristen Cares, MD, Department of Pediatric Gastroenterology, Children's Hospital of Michigan, 3901 Beaubien Blvd, Detroit, Michigan 48201. e-mail: kcares@dmc.org.

Authors' Contributions:
Kristen Cares: Conceptualization, methodology, data collection, writing, and editing. Ron Thomas: Methodology, data collection, data analysis, and editing. Amy Stolinski: Conceptualization, methodology, reviewing, and editing. Jacqueline Parker: Conceptualization, methodology, data collection, reviewing, and editing. Mohammad El-Baba: Conceptualization, methodology, data collection, review, and editing.

Conflicts of Interest:
The authors disclose no conflicts.

Funding:
The Children's Foundation (Grant #R2-2019-11A).

Ethical Statement:
This study was approved by the Institutional Review Board (IRB) at Children's Hospital of Michigan. The approval number for this study is 19-09-1295. Informed consent was obtained from all participants and legal guardians prior to enrollment. The study was conducted in accordance with Declaration of Helsinki and all relevant ethical guidelines.

Data Transparency Statement:
Individual participant data will not be shared. All analytic methods and datasets generated during the study are available from the corresponding author upon request. Surveys are available publicly and referenced in the article.

Reporting Guidelines:
CONSORT.

Gastro Hep Advances, Volume 5

Supplemental information

Impact of Proactive Therapeutic Drug Monitoring on Infliximab Maintenance Therapy and Clinical Outcomes in Pediatric Inflammatory Bowel Disease: A Randomized Controlled Trial and Review of Literature

Kristen Cares, Ron Thomas, Amy Stolinski, Jacqueline Parker, and Mohammad El-Baba

Supplemental Table 1. Post Optimization Characteristics

Characteristics	Post-Optimization phase	Post-Maintenance SOC Group (n = 19)	Post-Maintenance pTDM Group (n=21)
Treatment optimization, n (%)	29 (58)	4 (21)	5 (24)
Type of optimization, n (%)			
IMM	1 (3)	0	1 (20)
Infliximab dose only	13 (44)	3 (75)	4 (80)
Infliximab frequency only	5 (17)	0	0
Infliximab dose + frequency	8 (28)	0	0
IMM + Infliximab dose	2 (7)	1 (25)	0
Mean number of optimizations per patient, n	1.1 ± 0.3	1.25 ± 0.5	1 ± 0
Reason for optimization, n (%)			
Symptoms only	1 (4)	1 (25)	1 (20)
Abnormal labs only	0	0	0
Symptoms + abnormal labs	0	1 (25)	0
Abnormal infliximab assay	17 (63)	0	3 (60)
Abnormal assay + symptoms	3 (11)	2 (50)	1 (20)
Abnormal assay + symptoms + labs	5 (18)	0	0
Weight gain	1 (4)	0	0

Supplemental Table 2. Characteristics Pre and Post Optimization phase

Characteristics	Pre-Op	Post-Op	Mean Diff/SE	95% CI	*p*
Weight (kg)	63.50±24.00	66.16±24.49	2.56±5.55	-0.79-4.44	**.006***
BMI (kg/m2)	23.11±8.26	24.90±7.47	1.79±4.53	0.23- 3.34	**.026***
Hemoglobin (g/dL)	13.04±1.45	12.92±1.49	0.12±0.84	-0.15-0.38	.391
Albumin (g/dL)	4.24±0.41	4.28±0.31	0.04±0.38	-0.16-0.08	.513
C-reactive protein (mg/dL)	5.51±6.02	5.33±3.34	0.18±6.62	-1.96-2.33	.863
ESR	12.38±11.78	11.08±9.07	1.30±9.58	-1.76-4.36	.396
PCDAI/PUCAI	9.13±9.53	6.31±6.88	2.82±5.46	1.06-4.56	**.002***
Clinical remission n (%)	21/40 52.5%	28/40 70.0%			.168
Dose infliximab (mg/kg)	7.81±2.38	9.24±2.06	1.43±2.55	0.61-2.24	**.001***
Frequency of infliximab (weeks)	7.63±0.77	6.90±1.00	0.75+0.96	0.42-2.24	**≤.001***
Abnormal infliximab assay, n (%)	24/40 60.0%	14/40 35.0%			**.043***
Infliximab trough conc (ug/dL)	6.04±3.82	9.31±3.58	+3.27±4.00	1.99-4.55	**≤.001***
Abnormal infliximab trough, n (%)	19/40 47.5%	1/40 2.5%			**≤.001***
Infliximab antibody (ng/mL)	58.03±90.99	49.88±70.04	8.15±73.08	-15.22-31.52	.485
Abnormal infliximab antibody, n(%)	18/40 45.0%	13/40 32.5%			.359
Patient QOL survey (scale 5-25, 5 = best health)	6.31±1.76	5.92±1.09	0.39±1.41	-0.07-0.84	.096
Patient well being scaling score (100 = best health)	88.10±17.15	89.95±11.01	1.85-14.16	2.68-6.38	.414
Parent reported QOL survey (scale 5-25, 5 = best health)	6.69±2.21	6.00±1.44	0.069±1.47	0.13-1.25	**.017***
Parent reported well being scaling score (100 = best health)	86.50±18.01	92.21±8.69	5.71±12.52	0.86-10.57	**.023***

Supplemental Table 3. Adverse Events

Event, n (%)	Optimization phase (N = 51)	Maintenance phase SOC (N = 18)	Maintenance phase TDM (N = 21)	Related to Infliximab
Perirectal abscess			1 (4.8)	N
Pharyngitis			1 (4.8)	U
Pilonidal cyst			2 (9.5)	N
Apraxia			1 (4.8)	N
Folliculitis	1 (2)			U
Depression	1 (2)	1 (5.5)		N
COVID	1 (2)			U
Neutropenia		1 (5.5)		N
Headache	1 (2)			U
Tremors		1 (5.5)		U
Psoriasis	1 (2)			Y

Y = yes, N = no, U = unknown
